# Genome-wide analysis of the XTH gene family and functional analysis of *DlXTH23.5/25* during early longan somatic embryogenesis

**DOI:** 10.3389/fpls.2022.1043464

**Published:** 2022-11-23

**Authors:** Xiangwei Ma, Yan Chen, Mengyu Liu, Xiaodong Xue, Xueying Zhang, Luzhen Xu, Zhongxiong Lai, Yuling Lin

**Affiliations:** Institute of Horticultural Biotechnology, Fujian Agriculture and Forestry University, Fuzhou, Fujian, China

**Keywords:** *D. longan*, xyloglucan endotransglucosylase/hydrolase (XTH), transcription factor, somatic embryogenesis, heat stress

## Abstract

**Introduction:**

Xyloglucan endotransglucosylase (XET)/hydrolase (XTH) is a cell wall-modifying protein that affects cell expansion and loosening of the cell wall.

**Results:**

This study focused on the regulatory mechanism of DlXTH genes during early somatic embryogenesis (SE) and the heat stress response in longan. Mining of the available D. longan genome sequence yielded 25 putative XTH genes. Transcript profiles based on RNA sequencing (RNA-seq) data showed that most of the 17 detected DlXTH genes were highly expressed in the embryogenic callus (EC) (8) and globular embryo (GE) (8), and 13 of them responded significantly to heat stress. The assay for transposase-accessible chromatin sequencing (ATAC-seq) data analysis showed that in terms of chromatin accessibility, 22 of the 25 DlXTH genes were open during early SE, and most of the peak DlXTH genes with transcription differences during early SE were associated with high levels of H3K4me1. The most differentially expressed genes, DlXTH23.5 and DlXTH25, were selected for analysis. According to subcellular localization and quantitative real-time PCR (qRT-PCR) analysis, DlXTH23.5/25, which encode cell membrane-localized proteins, were expressed at the highest level in the GE and significantly responded to heat stress. Dual-luciferase assays and transient transformation showed that the transcription factors (TFs) DlWRKY31, DlERF1, and DlERF5 might bind to the DlXTH23.5/25 promoters to activate gene transcription. Transient overexpression of TFs and DlXTH23.5/25 induced XET activity in Nicotiana benthamiana leaves. Under heat stress in the longan EC, the XET activities and expression levels of TFs and DlXTH23.5/25 were significantly increased, and a high concentration of XET might inhibit longan SE.

**Discussions:**

Thus, the regulatory network composed of DlXTH23.5/25 and its related TFs may regulate early longan SE and participate in the regulatory pathway of longan under heat stress via cell wall repair through the action of XET.

## Introduction

Xyloglucan is the most abundant type of hemicellulose polysaccharide in the primary cell walls of dicotyledons and monocotyledons of non-Gramineaceae and forms the main framework of the primary plant cell wall *via* cross-linking with cellulose, pectin polysaccharides, and extensin (Bacic et al, 1988). Xyloglucan endotransglucosylase/hydrolase (XTH) is a cell wall-modifying protein that can catalyze the hydrolysis or transfer of xyloglucan molecules and achieve cleavage or rearrangement of the main chain of xyloglucan in the cell wall (Du et al., 2010). Most *XTHs* cut and rejoin xyloglucan *via* the activity of XET (EC 2.4.1.207), whereas some *XTHs* hydrolyze xyloglucan *via* the activity of xyloglucan hydrolase (XEH; EC 3.2.1.151) (Rose et al., 2002; Baumann et al., 2007). Both types of *XTH* genes may affect cell expansion and loosen the cell wall (Nishitani and Vissenberg, 2006). Previous studies have shown that XTH enzymes strongly influence cell organization and tissue tension in plants (Chanliaud et al., 2004; An et al., 2009). *XTHs* contribute to the regulation of the resulting plant phenotype by altering the structural properties of the cell wall.

XTH is a key enzyme in the process of plant cell wall remodeling; it provides cell wall ductility without reducing the mechanical properties of the cell wall and supports cell volume growth driven by turgor pressure (Cosgrove, 2005; Wang et al., 2021). Multigene families of *XTHs* have been identified in a wide variety of plant species, including *Arabidopsis* (Ryusuke and Kazuhiko, 2001), rice (Yokoyama, 2004), tomato (Miedes and Lorences, 2009), soybean (Li et al., 2018), and barley (Fu et al., 2019). The *XTH* gene family is involved in various physiological processes in plants, especially in abiotic stress responses and cell elongation. The reduction in the xylan content in the *xth31* mutant of *Arabidopsis* reduces the ability of mutant *xth31* to absorb aluminum ions, thereby improving the tolerance of plants to aluminum ions (Kochian, 1995). *XTH6* and *XTH24* of apple jointly respond to drought and salicylic acid (SA) (Long, 2018). The homologous genes in pepper, *pCaXTH1*, *pCaXTH2*, and *pCaXTH3*, were simultaneously induced by drought, high salt, and low temperature, indicating that they respond to abiotic stress (Cho et al., 2006a). Transcription of the *VaXTH1* and *VaXTH2* genes during phloem formation in azuki bean supports their involvement in plant development (Takuma et al., 2003). Bourquin et al. (2002) observed that an XTH protein was involved in the formation of secondary cell walls in vascular tissues of poplar. Transcription factors (TFs) are proteins that bind to specific DNA motifs in the promoter regions of target genes to regulate their transcription (Zhu, 2016; Horstman et al., 2017). In previous studies, *XTH* genes were shown to be regulated by TFs. *AtXTH19* and *AtXTH2* are regulated by the TF *BES1* downstream of brassinolide (Br) signaling (Xu et al., 2020). TF *ANAC017* regulated the expression of *AtXTH31* by directly binding to the promoter region, and in *anac017* mutants, the overexpression of *XTH31* resulted in an aluminum tolerance phenotype (Ye et al., 2022). The TF *WRKY47* could directly regulate the expression of *XTH17*, and increasing the expression of *XTH17* rescued aluminum tolerance and root growth in the *wrky47-1* mutant (Chun et al., 2020).

Longan (*Dimocarpus longan* Lour.) belongs to Sapindaceae *Dimocarpus*, and it is a tropical and subtropical evergreen fruit tree and an economically important crop (Mei et al., 2014). Moreover, longan also has multiple pharmacological uses, including large amounts of polyphenols, and has preventive effects against inflammation, cancer, and cardiovascular diseases (Zhang et al., 2012). It is difficult to sample longan early embryos under natural conditions, and the consistency of the materials is poor, which represents a great obstacle to the development of longan embryo systems. The somatic embryogenesis (SE) system established for longan was a model system for the study of SE in woody plants (Chen et al., 2018). Embryogenesis is a key process in the development of higher plants, but it is difficult to obtain and observe plant embryos. SE is very similar to the process of embryo development, so SE has become a good substitute for studying the mechanism of plant embryogenesis (Radoeva et al., 2019). The *XTH* gene family also plays important roles during SE. XET activity increased rapidly in heart-shaped, torpedo-shaped, and cotyledon-shaped embryos in carrots (Hetherington and Fry, 2017). In cucumber, the expression of *CsXTH1* and *CsXTH3* increased significantly during SE, and the highest expression was mainly in the cotyledon primordium (Malinowski et al., 2010). Robert et al. (2018) found that cells in the XET-enriched region were strongly extended during cucumber SE, with a decrease in XET activity in cotyledon-shaped embryos and enhanced XET activity in torpedo-shaped embryos. Next-generation sequencing (Lin et al., 2017) and third-generation sequencing (SRR17675476) of the longan “HongHeZi” (“HHZ”) genome were beneficial for studying the regulatory roles of *DlXTHs* during early longan SE. However, analysis of *XTH* genes in longan has not been carried out.

In this study, we identified 25 longan *XTH* genes and analyzed their phylogenetic relationships, chromosomal distributions, motif compositions, *cis*-acting elements, and expression patterns during early longan SE. To establish the roles of *DlXTH* genes in the response to stresses, we evaluated their response to heat/cold treatments. *DlXTH23.5/25* were screened from transcriptome data, and their promoter-binding TFs were predicted. We investigated the regulatory effects of TFs on *DlXTH23.5/25* by luciferase assays and transient expression of TFs in longan. In addition, the responses of TFs and *DlXTH23.5/25* to heat stress and their effects on XTH enzyme activity were detected. The results will expand our understanding of the *XTH* family in longan and further reveal the mechanism of the TF-modulated *DlXTH23.5/25* gene during early longan SE.

## Materials and methods

### Plant materials

Early SE cultures of longan, which involved the embryogenic callus (EC), incomplete compact pro-embryogenic cultures (ICpECs), and globular embryo (GE), were obtained as previously described (Lai et al., 1997). Taking the “HHZ” longan EC as the primary material, the longan EC proliferated for 20 days and was transferred to Murashige and Skoog (MS) medium and placed in 25°C and 35°C incubators in the dark. After 6, 9, and 12 days of treatment, the materials were collected and stored in liquid nitrogen at -80°C until use.

### Identification and phylogenetic tree of the *DlXTH* gene family

The protein sequences of *Arabidopsis* XTH family members were downloaded from the online website https://www.arabidopsis.org, and the AtXTH sequence was used as the probe sequence (e-value <0.001). The “HHZ” longan genome database from the National Center for Biotechnology Information (NCBI) Sequence Read Archive (SRA) database (SRR17675476) was used for homology alignment, and 25 candidate longan XTH family sequences were screened. Blast comparison was performed on the Arabidopsis information resource (TAIR) online website, and members of the DlXTH family were named with reference to *Arabidopsis*. The XTH conserved domain (PF06955) was downloaded from Pfam (http://pfam.xfam.org/). The basic physicochemical properties of the DlXTH protein were analyzed using the Expasy Protparam (https://web.expasy.org/protparam/). The phylogenetic tree was built with the neighbor-joining (NJ) method and 1,000 bootstrap replicates in MEGA X (v. 11.0.13) (Kumar et al., 2018).

### Chromosomal localization and synteny analysis

Chromosomal localization information was extracted from the GFF file, and the results were visualized with Tbtools. MCScanX (Wang et al, 2013) was used for the identification of syntenic gene pairs, and BLASTP results and gene location information were used for the next step input. Tbtools (Chen et al., 2020) was used to identify proximal, dispersed, tandem, and segmental/whole-genome duplications (WGDs) of *DlXTH* family genes. The results were used in Tbtools (Circle gene view).

### Analysis of conserved motifs, gene Structure, and *cis*-acting regulatory elements

Conserved motif analysis of DlXTH proteins was performed with Multiple Em for Motif Elicitation (MEME) (https://meme-suite.org/meme/) using the default parameters, and the maximum number of pattern parameters was set to 20. The gff and genome files of longan were used to construct gene structures with Tbtools (Visualize Gene Structure). A 2,000-bp sequence upstream of the transcription start site of genes in the *DlXTH* gene family was extracted from the longan genome file, and PlantCARE (http://bioinformatics.psb.ugent.be/webtools/plantcare/html/) was used to predict *cis*-acting elements. The results of the prediction were visualized with Tbtools.

### Analysis of the DlXTH gene family by RNA sequencing, assay for transposase-accessible chromatin sequencing, and chromatin immunoprecipitation sequencing

To elucidate the gene expression profile of the *DlXTH* gene family, RNA-seq data during early longan SE were available in the NCBI SRA repository under accession code SRR21979789, SRR21979788, and SRR21979787 (EC); SRR21979786, SRR21979785, and SRR21979784 (ICpEC); and SRR21979783, SRR21979782, and SRR21979781 (GE). Different temperature treatments are available in the NCBI SRA repository under accession code SRR21921625, SRR21921624, and SRR21921623 (15°C); SRR21921622, SRR21921621, and SRR21921620 (25°C); and SRR21921619, SRR21921618, and SRR21921617 (35°C). The Tbtools tool (log10 normalization) was used to display the heatmap of *DlXTH* gene expression based on RNA-seq data by hierarchical clustering and Euclidean distance analysis. Data from the assay for transposase-accessible chromatin sequencing (ATAC-seq) (SRR18028214, SRR18028213, and SRR18028212) and chromatin immunoprecipitation sequencing (ChIP-Seq) (SRR18035255, SRR18035254, and SRR18035253) assays during early longan SE (EC, ICpEC, and GE) were downloaded from the NCBI SRA database.

### Dual-luciferase reporter assays in tobacco leaves

The *DlXTH23.5/25* promoter (2,000 bp upstream of ATG) was amplified and cloned into pGreenII 0800-LUC to generate the reporter construct, while the TFs *DlWRKY31_Dlo003382*, *DlERF1_Dlo019949*, and *DlERF5_Dlo031758* were cloned into the CaMV35S vector as the effector (CaMV35S-*DlWRKY31/DlERF1/DlERF5*). The effector and reporter constructs were transformed into *Agrobacterium tumefaciens* strain GV3101 and coinfected into *Nicotiana benthamiana* leaves by agroinfiltration, as previously described (Sheludko et al., 2007). The transcriptional activity of the TFs *DlWRKY31*, *DlERF1*, and *DlERF5* was indicated by the ratio of LUC/REN using a Dual-Luciferase Reporter Gene Assay Kit (Yeasen, Shanghai, China) on a Multiskan Spectrum microplate spectrophotometer (BioTek, CA, USA). At least three biological replicates were examined for each sample.

### Isolation and transformation of longan protoplasts

Protoplasts were isolated from the longan EC, which was grown for approximately 15 days. The longan EC was mixed with 10 ml of enzyme solution, including 5 ml of MS liquid medium containing 20.0 g/L sucrose and 1.0 mg/L 2,4-D, and 5.0 ml of enzymatic hydrolysis solution, including 0.93 M/L mannitol, 1 M/L CaCl_2_·2H_2_O, 0.005 g bovine serum albumin (BSA), 0.1 M/L MES, 0.06 g macerozyme R10, and 0.12 g cellulase R10, brought up to 5 ml with water, and digested for 12 h at 25°C in the dark. A cell strainer (40-μm diameter) was used to filter the sample, which was washed with 8% mannitol twice and then centrifuged (500 g/min) for 5 min at 28°C. The sample was resuspended in MMG (0.93 M/L mannitol, 0.5 M/L MgCl_2_·6H_2_O, and 0.1 M/L MES and brought up to 10 ml with water), and the vitality of the protoplasts was observed with a microscope. The concentration of protoplasts was adjusted to 1,000–5,000 protoplasts/μl.

Transient transformation of protoplasts was performed according to the polyethylene glycol (PEG)-based method (Tang et al., 2013). Two hundred microliters of protoplast suspension was added to a 1.5-ml centrifugation tube, after which 5 µg recombinant plasmid with 15 µl sterile ddH_2_O was added. An equal volume of PEG solution was added (10 ml PEG solution included 0.73 M/L mannitol, 4 g PEG4000, and 1 M/L CaCl_2_•2H_2_O, and water was added to reach 10 ml). The prepared solution was mixed gently and then allowed to stand for 5.5 min before 440 µl W5 buffer was added to stop the reaction. After centrifugation (300 rcf/min for 1 min), the sample and the supernatant were collected. After adding 440 µl W5 buffer, the sample was centrifuged again, and the abovementioned steps were repeated. The transformed protoplasts were resuspended in 0.5 ml W5 buffer and transferred to a 24-well plate at 25°C for 24 h on a shaker (50 r/min).

### Transient transformation of the longan EC and tobacco

*DlWRKY31*, *DlERF1*, and *DlERF5* recombinant plasmids with GUS tags were transferred into an *A. tumefaciens* strain (GV3101) by the freeze−thaw method. Activated *A. tumefaciens* was transferred into 20 ml of Luria-Bertani (LB) and cultured at 28°C for 16 h with shaking at 200 rpm. The longan ECs, which were grown for approximately 15 days, were transferred into the bacterial liquid of *A. tumefaciens* (OD600 = 0.6–0.8). After 30 min of cocultivation, the infected ECs were transferred to MS solid medium containing 30 g/L sucrose for 3 days. The infection solution of transient tobacco included 500 mM MES, 100 mM Acetosyringone (AS), and 100 mM MgCl_2_.

### Subcellular localization analysis

The full-length coding sequences of *DlXTH* genes (DlXTH23.5/25) without stop codons were amplified with primers (**Supplementary Material 1**) and cloned into the pCAMBIA1302-35S-GFP vector. pCAMBIA1302-35S-DlXTH23.5:GFP and pCAMBIA1302-35S-DlXTH25:GFP were transiently expressed in tobacco (*N. benthamiana*), whose leaves were infiltrated by *Agrobacterium*. The tobacco plants were kept in a dark environment at 26°C for 3 days. Plasmolysis of tobacco leaves was induced with sucrose solution (10 ml sucrose solution included 7 g sucrose). Confocal laser scanning microscopy (FV1200, Olympus) was used to analyze the fluorescence signals of GFP.

### RNA extraction and quantitative real-time PCR analysis

Total RNA was extracted from longan SE samples and protoplasts using TRIzol up reagent (TransGen, China), and cDNA synthesis was performed using the SMART™ RACEcDNA Amplification Kit TransScript RNA First-Strand cDNA Synthesis SuperMix (YEASEN, China). A 10-fold dilution of cDNA was used as a template for amplification, and qRT−PCR detection was performed on a Roche Light Cycler 480 instrument. Beta-actin (ACTB) was used as an internal reference gene for qRT−PCR of early longan SE samples, and ubiquitin (UBQ) was used as an internal reference gene for qRT−PCR of the longan EC and transient transformation of protoplasts (**Supplementary Material 1**). The relative expression of *DlXTH* was calculated by the 2^-ΔΔCT^ method, and the data were imported into SPSS software to analyze significant differences. Different letters representing significant differences were assessed by Duncan test (Student’s t-test, *P < 0.05, **P < 0.01), and GraphPad Prism 9 was used to generate graphs.

## Results

### Identification, classification, and phylogenetic analysis of the XTH genes in longan

The genome of *D. longan* was scanned to identify *XTH* family genes using Basic Local Alignment Search Tool (BLAST). The amino acid (aa) sequences of the known *XTH* members in *Arabidopsis* were used as queries. Twenty-six putative DlXTH protein sequences were identified. Simple Modular Architecture Research Tool (SMART) and biosequence analysis using profile hidden Markov models (HMMER) (https://www.ebi.ac.uk/Tools/hmmer/) were used to confirm the existence of the conserved XTH domain (PF06955), and redundant sequences were removed. We finally obtained 25 genes in the longan XTH family. For annotation of the 25 identified longan *XTH* genes, the *Arabidopsis* nomenclature system was used, with numbers representing the highest sequence similarity with the corresponding AtXTH ortholog (**Table 1**). The number of aa in the DlXTH protein sequences ranged from 3,299 aa (DlXTH23.6) to 5,612 aa (DlXTH30.2), with an average length of 4,551.75 aa. The molecular weights (MWs) of the predicted encoded proteins varied from 23.96 kDa (DlXTH23.6) to 40.68 kDa (DlXTH30.2), with an average of 33.08 kDa, and the theoretical isoelectric point ranged from 4.91 to 9.47. All of the DlXTHs were categorized as unstable proteins because their instability index was greater than 40 (52.72~73.4). All of the DlXTH proteins had negative grand average hydropathicity (GRAVY) scores, indicating that all of the DlXTH proteins were hydrophilic (**Table 1**).

**Table 1:** Analysis of the basic parameters of the *DlXTH* family.

Gene ID (PacBio+Illumina+Hi-C)	Gene ID (Illumina)	Gene name	Size (aa)	Molecular Weight/kD	Isoelectric Point (PI)	Instability coefficient	Hydrophilicity
Dlo000037	/	DlXTH26	4,574	33,062.35	7.7	44.94	-0.351
Dlo001347	Dlo_024538.1	DlXTH23.1	4,361	31,721.32	5.38	36.16	-0.405
Dlo001351	Dlo_003713.1	DlXTH23.2	4,382	31,881.54	5.53	38.64	-0.399
Dlo001352	Dlo_003712.1	DlXTH22	4,455	32,417.05	5.96	33.66	-0.482
Dlo001353	Dlo_003711.1	DlXTH25	4,587	33,149.39	8.87	35	-0.444
Dlo001354	Dlo_003708.1	DlXTH23.3	4,352	31,664.29	5.66	35.98	-0.413
Dlo001355	Dlo_003707.1	DlXTH23.4	4,365	31,755.45	5.77	33.17	-0.395
Dlo001356	Dlo_003703.1	DlXTH23.5	4,357	31,636.42	5.73	39.41	-0.371
Dlo002170	Dlo_003397.1	DlXTH15	4,553	32,882.32	9.12	45.21	-0.374
Dlo011689	Dlo_030734.1	DlXTH2.1	4,115	30,019.46	9.08	33.85	-0.777
Dlo011693	Dlo_030731.1	DlXTH2.2	4,217	30,942.14	6.89	30.15	-0.801
Dlo012834	Dlo_017323.1	DlXTH23.6	3,299	23,955.76	4.91	32.39	-0.409
Dlo016076	Dlo_000475.1	DlXTH6	4,515	32,702.96	7.04	37.77	-0.345
Dlo017787	Dlo_016241.1	DlXTH27	5,255	38,148.73	6.49	45.45	-0.387
Dlo018698	Dlo_018550.1	DlXTH33	4,619	35,538.13	6.86	49.4	-0.232
Dlo019221	Dlo_028730.1	DlXTH30.1	4,639	33,843.92	5.13	33.63	-0.36
Dlo021366	Dlo_025998.1	DlXTH8	4,734	34,688.83	5.04	36.48	-0.543
Dlo023720	Dlo_017030.1	DlXTH10	4,705	33,925.36	8.89	32.8	-0.434
Dlo026127	Dlo_030117.1	DlXTH32.1	4,544	32,849.82	6.37	50.76	-0.416
Dlo027186	Dlo_008109.1	DlXTH9	4,547	33,003.09	5.49	35.47	-0.343
Dlo027460	Dlo_011343.2	DlXTH5.1	4,712	34,125.48	8.65	42.63	-0.471
Dlo028271	Dlo_018886.1	DlXTH30.2	5,612	40,679.64	8.58	52.12	-0.528
Dlo029483	Dlo_025579.1	DlXTH32.2	4,695	33,967.59	9.47	41.05	-0.467
Dlo032646	Dlo_002033.1	DlXTH5.2	4,722	34,191.9	8.76	42.11	-0.451
Dlo032716	Dlo_028730.1	DlXTH30.3	4,683	34,189.28	4.95	34.8	-0.328

**Figure 1:**
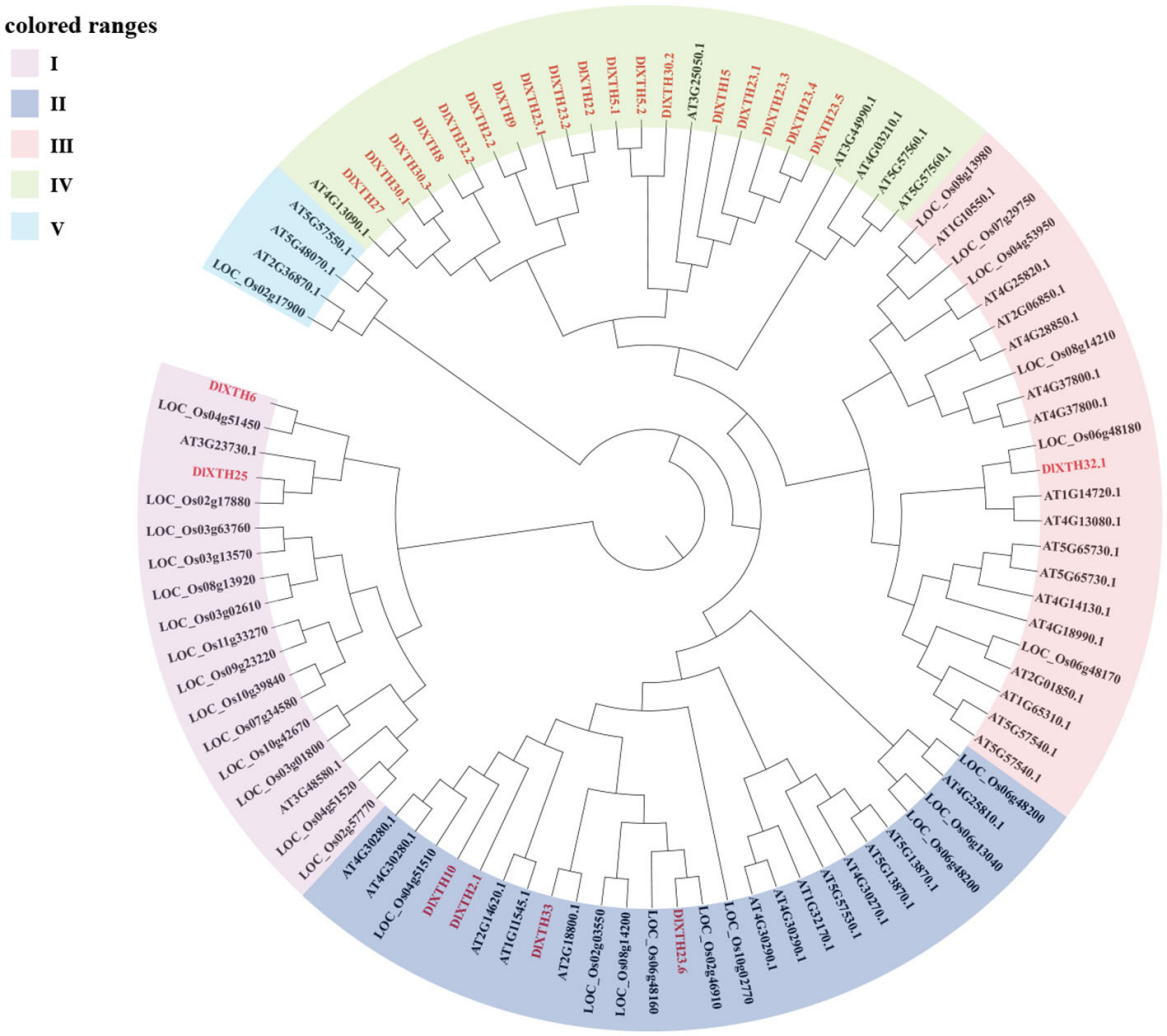
Phylogenetic tree analysis of xyloglucan endotransglucosylase/hydrolase (XTH) proteins from longan, *Arabidopsis*, and *Oryza*. The five XTH subgroups are represented by different colors: purple for subgroup I, dark blue for subgroup II, pink for subgroup III, green for subgroup IV, and light blue for subgroup V.

The phylogenetic tree of the *XTH* gene family contains *XTH* gene members from three species: *D. longan*, *O. sativa*, and *A. thaliana*. The phylogenetic tree was constructed using the NJ method and tested by 1,000 bootstrap replicates (**Figure 1**). The results suggested that the *XTH* genes could be categorized into five subgroups (Clades I~V); most *XTH* genes (72%) were distributed in Clade IV, and these *XTH* genes were clustered with *Arabidopsis* members. In Clade I, there were two DlXTH proteins (DlXTH6/25). Clade II contained four DlXTH proteins (DlXTH10/2.1/23.6/33). Clade III was the smallest, containing only one DlXTH protein (DlXTH32.1). The XTH protein in longan is more similar to that in dicotyledons than to that in monocotyledons, indicating that XTH proteins in longan are more closely related to those in dicotyledons than to those in monocotyledons.

### Gene structure and conserved motif identification of DlXTHs

To further understand the evolutionary relationships of the 25 *DlXTH* genes, we identified structural features and motifs of the *DlXTH* genes, including the locations of exons, introns, and conserved motifs (**Supplementary Figure S1**). According to our statistics, the number of introns in the *DlXTH* family genes varied from 0 to 4, and 15 DlXTHs contained Untranslated Regions (UTRs) (**Supplementary Figure S1A**). In addition, 10 conserved motifs of DlXTH proteins were identified by MEME analysis **Supplementary Figure S1B**). Motifs 1–5 and 9 were detected in all DlXTH proteins except DlXTH23.6/33, which lacked motifs 5 and 9. Motif 6 was not detected in one member of Clade II (*DlXTH23.6*) and three members of Clade IV (DlXTH2.2/32.1/32.2) but was detected in all of the remaining members.

### Chromosomal localization and synteny analysis of *DlXTHs*

**Figure 2:**
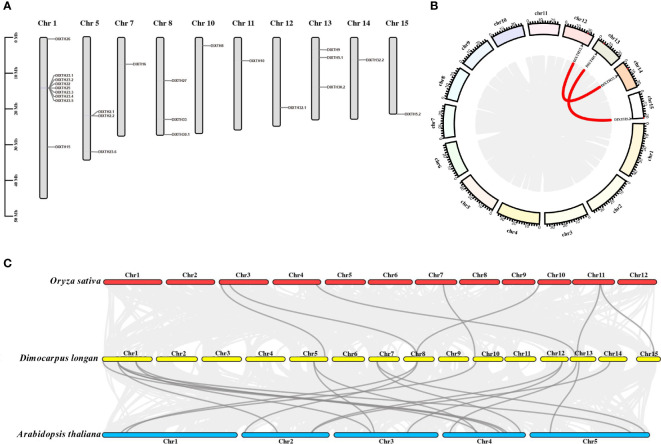
Chromosome location, gene duplication event collinearity, and synteny of *DlXTH* genes. **(A)** Chromosomal locations of 24 *DlXTH* genes. **(B)** Red points along the circumference indicate the positions of *DlXTH* genes on chromosomes. The red lines inside the circle represent collinearity relationships among *DlXTH* genes. **(C)** Synteny analysis of *xyloglucan endotransglucosylase/hydrolase (XTH)* genes between longan and *Arabidopsis* or *Oryza*. Dark gray lines indicate collinear blocks of *XTH* genes within the longan, *Arabidopsis*, and *Oryza* genomes.

According to the gene locus information, the 25 *DlXTH* genes were unevenly distributed on 10 of the 15 chromosomes of longan (**Figure 2A**), and nine *DlXTH* genes were distributed on Chr1. Chromosomes 5, 8, and 13 had three genes, and the smallest numbers of genes were found on chromosomes 7, 10, 11, 12, 14, and 15 (one gene each). As shown in **Figure 2B**, we found that the *DlXTH* gene was located in the tandem cluster of Chr1, which contained seven consecutively aligned members (DlXTH23.1/23.2/22/25/23.3/23.4/23.5).

The results of collinearity analysis showed that there were two pairs of segmental duplication events in longan *DlXTHs*, corresponding to *DlXTH32.1/32.2* and *DlXTH5.1/5.2* (**Figure 2B**). The results indicated that some *DlXTHs* were probably generated by gene segmentation or tandem duplication, which may have been conducive to the evolution and expansion of the *DlXTH* gene family. To determine the evolutionary rates and selective pressures among the *DlXTH* genes and their duplicated genes, we used Ka (non-synonymous), Ks (synonymous), and the Ka/Ks ratio for estimations. In general, Ka/Ks < 1 represents purifying selection, Ka/Ks = 1 represents neutral evolution, and Ka/Ks > 1 represents positive selection. We calculated the selective pressures of each duplicated gene pair, and the Ka/Ks values are shown in **Supplementary Material 2**. Interestingly, the Ka/Ks values of all of the *DlXTH* gene pairs were less than 1, which indicated that the *DlXTH* gene family underwent strong purifying selection and demonstrated that this family was conserved during longan domestication. The collinearity analysis between longan and *Arabidopsis* or rice showed that six *DlXTH* homolog genes appeared in the last five chromosomes of rice (**Figure 2C**), but 11 *DlXTHs* had corresponding paralogous genes on nine chromosomes in *Arabidopsis*. The relationship between longan and *Arabidopsis* is closer than that between longan and rice.

### Characterization of *cis-*acting elements in *DlXTH* gene promoters

To understand the transcriptional regulation of *DlXTH* genes, we analyzed the 2.0-kb upstream promoter region of the *DlXTH* gene using the PlantCARE tool to predict potential *cis*-acting elements (**Supplementary Figure S2**). Many *DlXTH* genes presented elements associated with plant hormones [auxin, abscisic acid (ABA), SA, methyl jasmonate (MeJa), and gibberellic acid (GA)] and stress responses (drought inducibility and low temperature). In terms of hormone response elements, 19 *DlXTHs* contained gibberellin response elements, 16 *DlXTHs* contained methyl jasmonic acid and salicylic acid response elements, 12 *DlXTHs* contained gibberellin response elements, and four *DlXTHs* contained auxin response elements. The number of hormone elements present in *DlXTH* members varied, and it was speculated that *DlXTH* family members respond to different hormones to different degrees. These results indicated that *DlXTH* genes play an important role in complex hormone regulation and stress networks, which may be involved in multiple stress responses and hormonal regulation.

### Expression analysis of *DlXTH* genes during early SE and under different temperature stress conditions

**Figure 3:**
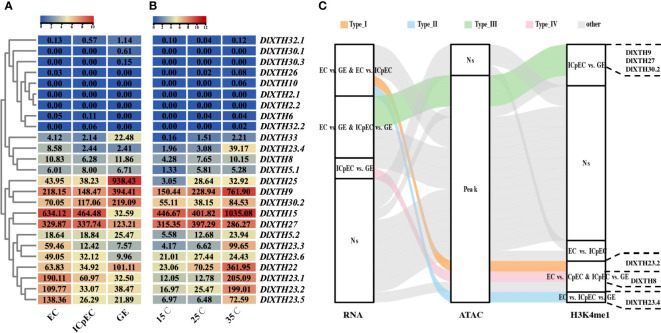
Expression profile during early somatic embryogenesis (SE) and under different temperature treatments; *DlXTH* is differentially accessible. **(A, B)** The remaining clusters based on expression analyses of *DlXTH* using previously published transcriptome data from longan early SE and different temperature treatments. **(C)** Alluvial diagram of differentially accessible *DlXTH* genes divided into five types (types I–IV and other). Types I–IV represent differentially accessible *DlXTH* genes among the RNA, assay for transposase-accessible chromatin sequencing (ATAC), and H3K4me1 databases. The other letters represent no differences among the three databases. The right panel shows representative genes of each type.

To investigate the biological roles of the *DlXTH* genes, analysis was performed based on a previous early SE RNA-seq dataset. Among these genes, there were eight *DlXTHs* (DlXTH26/2.1/2.2/6/30.1/10/32.2/30.3), and almost no expression was detected (**Figure 3A**). Of the remaining 17 genes, eight, one, and eight *DlXTH* genes were preferentially expressed in the EC, ICpEC, and GE, respectively, suggesting that they may function during early SE. In general, most of the *DlXTH* genes (*DlXTH22/8/32.1/30.2/25/5.2/33/9*) had higher expression levels in the GE than in other stages (**Figure 3A**), suggesting that these genes may play an important role in promoting the process of early SE. Notably, the six members annotated as *DlXTH23* were highly expressed in the EC stage. Except for *DlXTH23.6*, the remaining members were located in the tandem cluster of Chr1. The results showed that *DlXTH23* may be involved in maintaining the EC stage, and the functions of multiple members were redundant.

Previous studies have shown that some *XTH* genes are involved in various abiotic stress responses in plants (Cho et al., 2006a; Du et al., 2021). The expression patterns of *DlXTHs* were further studied under different temperature conditions by analyzing the RNA-seq data for the longan EC (**Figure 3B**). The results showed that the expression levels of most *DlXTH* genes varied obviously under heat and cold stress conditions. Of these genes, 13 and two were upregulated under the high-temperature and low-temperature treatments, respectively. It was inferred that *DlXTHs* had a stronger response to high temperature than to low temperature. Interestingly, the five *DlXTH23* members (except *XTH23.6*) not only were highly expressed in the EC but also significantly responded to high temperature in the longan EC. These results further demonstrate the similar functions of *DlXTH23* members during early SE.

ATAC-seq assays are an important part of epigenetic analysis and are widely used in the study of protein−DNA interactions and chromatin accessibility (Qiu et al., 2021). To further understand the changes in chromatin accessibility of the *DlXTH* family during early SE, ATAC-seq data were analyzed and showed that except for *DlXTH2.1*, *DlXTH32.1*, and *DlXTH30.3*, the chromatin accessibility of other *DlXTH* genes was open during the early stage of SE (**Figure 3C**). H3K4me1 is an epigenetic modification of the DNA packaging protein histone H3. This marker indicates monomethylation of the fourth lysine residue of histone H3 protein and is commonly associated with gene enhancers (Hoffman et al., 2010). However, when the H3K4me1 chromatin immunoprecipitation assays were combined with sequencing datasets, most of the peaks of *DlXTH* gene transcription differences during early SE were associated with high levels of H3K4me1 (**Figure 3C**). Interestingly, we found that *DlXTH9*, *DlXTH27*, *DlXTH30.2*, *DlXTH23.2*, *DlXTH8*, and *DlXTH23.4* had differential peaks of H3K4me1 binding and were differentially expressed, and chromatin accessibility increased during early SE (**Figure 3C**). In summary, transcription levels, chromatin accessibility, and H3K4me1 modifications have an impact on the mechanisms of *XTH* family involvement in early SE cell wall modifications.

### Transcription factors target the *DlXTH23.5/25* promoters and activate their transcription

**Figure 4:**
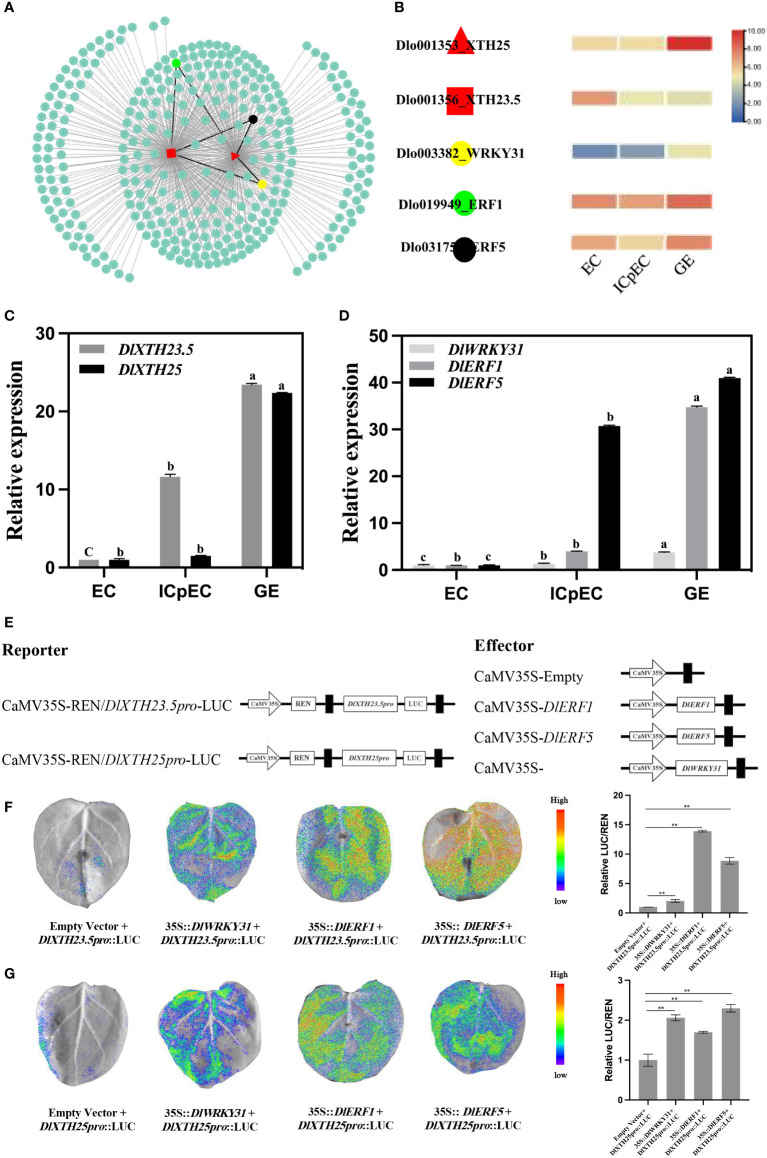
Transcription factors (TFs) regulate *DlXTH23.5* and *DlXTH25* expression. **(A)** TFs in the *DlXTH23.5* and *DlXTH25* relationship prediction diagram. **(B)** Expression profile of TFs during early somatic embryogenesis (SE). **(C)** Expression profiles of *DlXTH23.5* and *DlXTH25* during early SE by qRT−PCR. **(D)** Expression profiles of TFs (*DlWRKY31*, *DlERF1*, and *DlERF5*) during early SE by qRT−PCR. The data are expressed as the mean ± SD (n = 3). Different letters indicate significant differences. **(E)** Dual-luciferase assays of *DlXTH*-activating and candidate gene promoters in tobacco leaves. **(F, G)** Left: Image of firefly luciferase luminescence signals for the indicated reporter, and effectors were injected into tobacco leaves. Right: Relative reporter activity (LUC/REN) in tobacco leaves expressing the indicated reporters and effectors (Student’s t-test, **P < 0.01).

Since *DlXTH23.5* was downregulated more than 5-fold (high expression in the EC stage) and *DlXTH25* was upregulated more than 23-fold (high expression in the GE stage) during early SE (**Figure 3A**), their function during early longan SE attracted further interest. TFs regulate gene expression by binding to specific DNA sequences (Wittkopp and Kalay, 2012; Lambert et al., 2018; Guo et al., 2019). To understand the differences in *DlXTH23.5/25* expression, the upstream 2-kb sequence of *DlXTH23.5/25* was used as a TF-binding region by using PlantTFDB (http://planttfdb.gao-lab.org/prediction.php). A total of 329 TFs were predicted, of which 206 were shared by *DlXTH23.5* and *DlXTH25* (**Figure 4A**). Based on transcriptome data, *DlWRKY31*, *DlERF1*, and *DlERF5* were selected as candidate TFs for regulating *DlXTH23.5* and *DlXTH25*. In the RNA-seq dataset, the expression of the TFs *DlWRKY31*, *DlERF1*, and *DlERF5* was greatly increased in the GE stage (**Figure 4B**).

To preliminarily determine the regulatory effect of TFs on *DlXTH23.5/25* transcription, qRT−PCR was used to analyze the expression of three TFs and *DlXTH23.5/25* during early longan SE (**Figure 4C**). The expression levels of three TFs (*DlWRKY31*, *DlERF1*, and *DlERF5*) and *DlXTH23.5/25* gradually increased during early SE and were significantly upregulated in the GE stage (**Figure 4D**). Thus, TFs may enhance *DlXTH23.5/25* transcription by targeting their promoters. To test whether the TFs *DlWRKY31*, *DlERF1*, and *DlERF5* can activate the expression of *DlXTH23.5/25*, a luciferase (LUC) reporter assay was performed in tobacco leaves. Six constructs, *35S::DlWRKY31*, *35S::DlERF1*, and *35S::DlERF5*, were used as effectors, and *DlXTH23.5:LUC* and *DlXTH25:LUC* were used as reporters (**Figure 4E**). The relative *DlWRKY31*-induced firefly LUC/Renilla luciferase (REN) activity driven by the promoters of *DlXTH23.5* and *DlXTH25* increased 2.05-fold and 2.06-fold, respectively, compared with the control without *DlWRKY31* (**Figures 4F, G**). Moreover, transformation of *DlERF1* (13.91-fold) and *DlERF5* (8.88-fold) significantly enhanced the LUC activity driven by the promoters of *DlXTH23.5* (13.91-fold, 8.88-fold) and *DlXTH23.5* (1.76-fold and 2.30-fold). These results suggested that the TFs *DlWRKY31*, *DlERF1*, and *DlERF5* may target the *DlXTH23.5/25* promoters and activate their transcription.

### Subcellular localization and transient overexpression of transcription factors and *DlXTH23.5/25* enhanced XET activity

**Figure 5:**
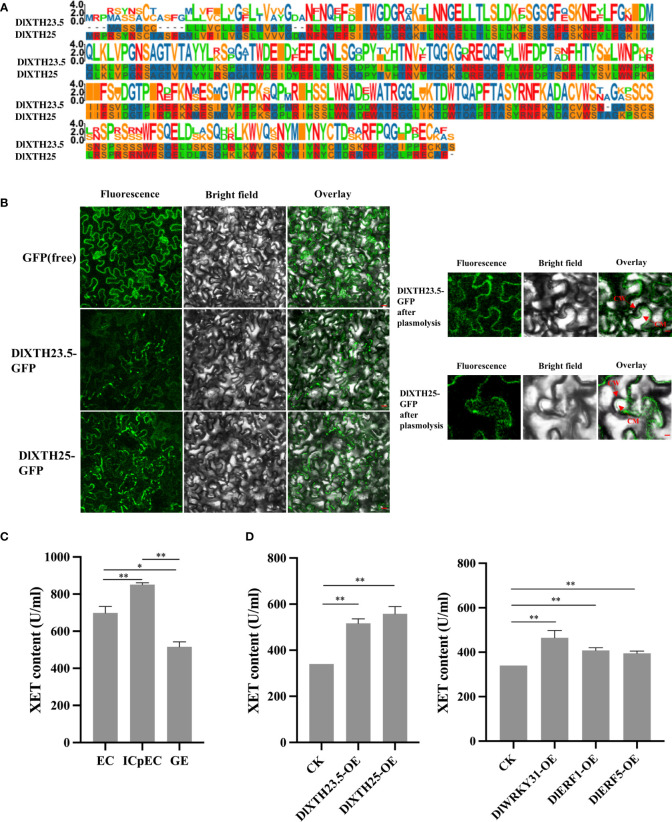
Transient expression of *DlXTH23.5/25* in tobacco and functional verification of *DlXTH23.5/25* with associated transcription factors (TFs). **(A)** Multiple sequence alignment of the DlXTH23.5/25 protein. **(B)** Subcellular localization of the DlXTH23.5–GFP and DlXTH25–GFP fusion proteins in tobacco leaves. Free GFP served as a control. CW, cell wall; CM, cell membrane. Scale bar = 20 μm. **(C)** Xyloglucan content during early somatic embryogenesis (SE) in longan. **(D)** Left: Xyloglucan content in wild-type and *DlXTH23.5/25*-overexpressing leaves. Right: Xyloglucan content in wild-type and *DlWRKY31*- and *DlERF1/5*-overexpressing leaves (Student’s t-test, *P < 0.05, **P < 0.01).

To meticulously analyze the structural features of the DlXTH23.5 and DlXTH25 proteins, multiple sequence alignment was performed. The results showed that DlXTH23.5 and DlXTH25 were highly conserved and contained the conserved diagnostic aa sequence motif DEIDFEFLG, which was the defining characteristic of XTH proteins (**Figure 5A**). To further analyze the function of DlXTH23.5 and DlXTH25, we investigated their subcellular localization in plant cells. The full-length coding sequence of DlXTH23.5 or DlXTH25 was fused with the GFP gene at the C-terminus and placed under the control of the 35S cauliflower mosaic virus (CaMV) promoter. The constructs and controls were introduced into *A. tumefaciens* GV3101/pSoup cells by transformation and infiltrated into tobacco leaves. Confocal microscopy showed signals at the cell membrane or cell wall for both DlXTH23.5-GFP and DlXTH25–GFP (**Figure 5B**). To distinguish potential localization on the cell wall or membrane, infiltrated plant cells were treated with a high concentration of sucrose solution to induce plasma–wall separation. The results showed that the DlXTH23.5–GFP and DlXTH25–GFP were detected exclusively in the cell membrane (**Figure 5B**), suggesting that DlXTH23.5/25 may be involved in cell wall modification.

XTHs are known to loosen cell walls, and their activity was measured to predict XTH-induced cell wall stress relaxation and elongation (Fry et al., 1992; Nishitani and Tominaga, 1992). First, the XET activity during early longan SE was determined using an enzyme-linked immunosorbent assay (ELISA), indicating the accumulation of XET activity in the ICpEC stage during early SE (**Figure 5C**). Then, the effects of *DlXTH23.5*, *DlXTH25*, and TF overexpression on XTH activities were analyzed in tobacco leaves (**Figure 5D**). Compared with that in the control, the XET activity of tobacco leaves overexpressing *DlXTH23.5* and *DlXTH25* was significantly increased. Similarly, XET activity was significantly increased in cells overexpressing the TFs *DlWRKY31*, *DlERF1*, and *DlERF5*. Based on these results, we speculated that *DlXTH23.5/25* regulation by TFs may be required for *XTH* accumulation and cell wall modification.

### Transient overexpression of transcription factors in longan promotes the expression of *DlXTH23.5/25*

**Figure 6:**
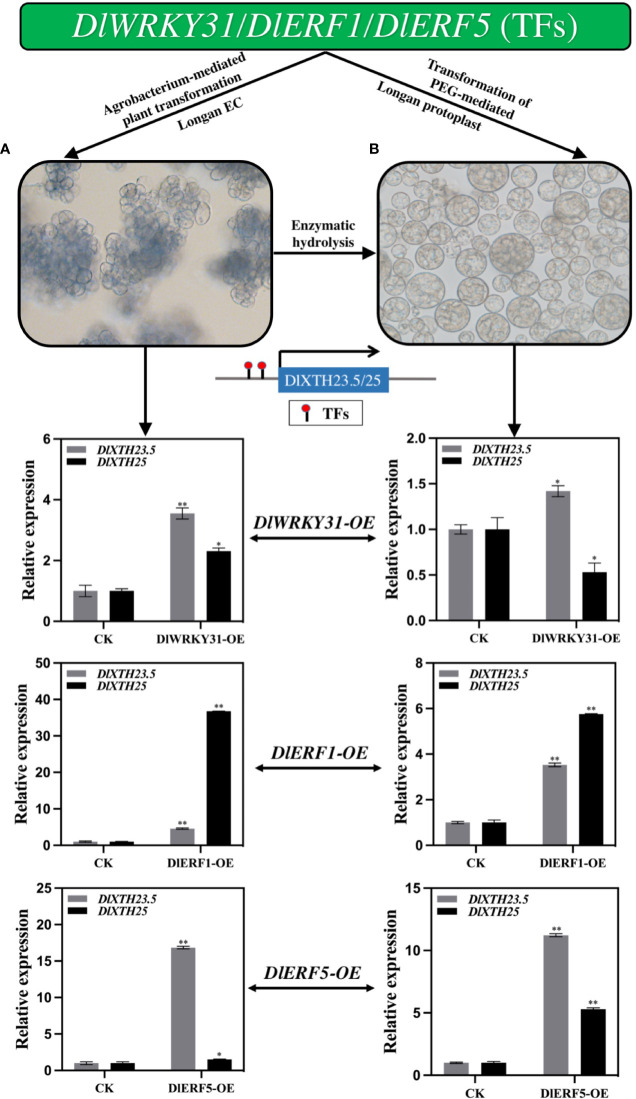
The expression profiles of *DlXTH23.5* and *DlXTH25* were regulated by transcription factors (TFs) under transient transformation in longan embryogenic calluses (ECs) and protoplasts. **(A)** Relative expression of TFs transiently transformed into longan ECs to regulate *DlXTH23.5* and *DlXTH25*. **(B)** Relative expression of TFs transiently transformed into longan protoplasts to regulate *DlXTH23.5* and *DlXTH25* (Student’s t-test, *P < 0.05, **P < 0.01).

To ascertain whether the TFs *DlWRKY31*, *DlERF1*, and *DlERF5* modulate *DlXTH23.5/25* expression in longan, we first investigated the expression of *DlXTH23.5/25* in the longan EC overexpressing the TFs *DlWRKY31*, *DlERF1*, and *DlERF5*. qRT−PCR analysis showed that after transient transformation with the TF *DlWRKY31*, the expression levels of *DlXTH23.5* and *DlXTH25* increased 2.31-fold and 3.55-fold, respectively (**Figure 6A**). Interestingly, the expression of *DlXTH25* in cells overexpressing *DlERF1* was significantly induced (36.76-fold), and the expression of *DlXTH23.5* was also increased by 4.58-fold (**Figure 6A**). Similarly, the expression of *DlXTH23.5* (16.85-fold) and *DlXTH25* (1.50-fold) was significantly increased in cells overexpressing the TF *DlERF5* (**Figure 6A**). Consistent with the above results, overexpression of the TFs *DlWRKY31*, *DlERF1*, and *DlERF5* in the longan EC significantly enhanced the expression of the *DlXTH23.5/25* gene.

To further determine the regulation of *DlXTH23.5/25* expression by TFs in longan, longan protoplasts were isolated for transformation. qRT−PCR analysis showed that the expression of *DlXTH23.5* was significantly induced in longan protoplasts overexpressing *DlWRKY31*, while *DlXTH25* expression was significantly downregulated (**Figure 6B**). In protoplasts overexpressing *DlERF1*, the expression levels of the *DlXTH23.5* and *DlXTH25* genes were 3.53-fold and 5.76-fold higher than those in the control, respectively (**Figure 6B**). Overexpression of *DlERF25* also elevated *DlXTH23.5* (11.22-fold) and *DlXTH25* (5.29-fold) expression (**Figure 6B**). In conclusion, the expression of *DlXTH23.5* and *DlXTH25* regulated by TFs in longan ECs and protoplasts was basically consistent. The TFs *DlWRKY31* and *DlERF5* were more likely to regulate *DlXTH23.5*, while the TF *DlERF1* was more likely to regulate *DlXTH25*. Collectively, the results suggest that the TFs DlWRKY31, DlERF1, and DlERF5 may promote expression by binding to the *DlXTH23.5/25* promoter region in longan.

### Transcription factors and *DlXTH23.5/25* responses to heat stress

**Figure 7:**
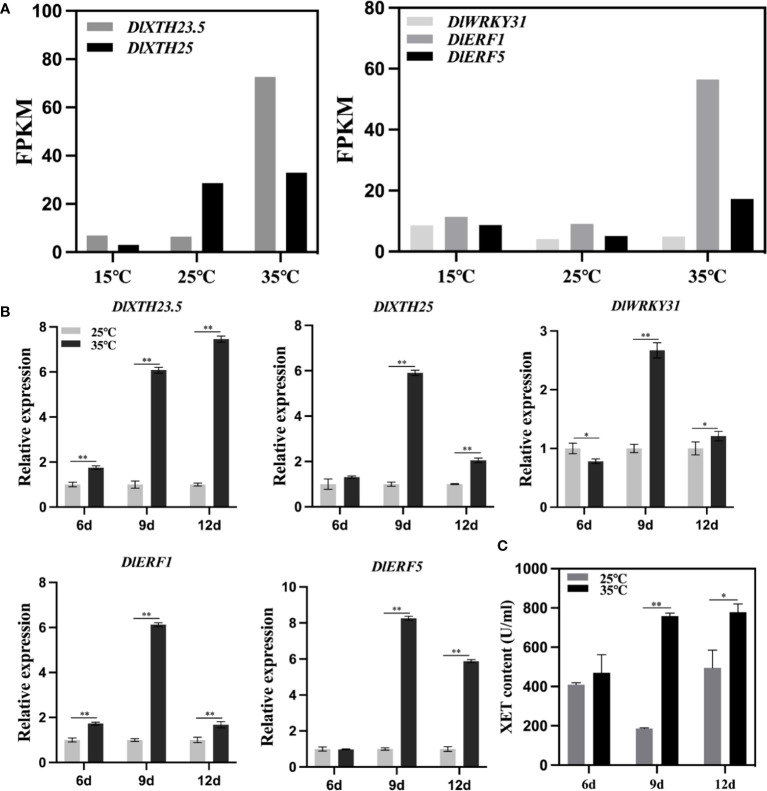
Expression pattern analysis of *DlXTH23.5* and *DlXTH25* and their associated transcription factors (TFs) under high-temperature stress. **(A)** Expression profiles of *DlXTH23.5* and *DlXTH25* and their associated TFs under different temperature treatments. **(B)** Relative expression of *DlXTH23.5* and *DlXTH25* and their associated TFs under heat stress on different days (Student’s t-test, *P < 0.05, **P < 0.01). **(C)** Xyloglucan content in longan embryogenic callus (EC) under heat stress on different days (Student’s t-test, *P < 0.05, **P < 0.01).

Based on the longan EC transcriptome sequencing data under temperature treatment, an expression heatmap of the *DlXTH* gene family under temperature treatment was generated (**Figure 3B**). The expression levels of *DlXTH23.5* and *DlXTH25* at high temperature were higher than those at low temperature, indicating that these two *DlXTH* genes responded to high-temperature stress (**Figure 7A**). TF *DlERF1* and *DlERF5* also had the same response to high-temperature stress. However, the expression of the TF *DlWRKY31* gene at high temperature was lower than that at low temperature, indicating that the TF *DlWRKY31* was responsive to low-temperature stress (**Figure 7A**).

To further explore the response of *DlXTH23.5/25* and related TFs to heat stress, we treated longan ECs at 35°C for different durations. The results showed that the expression of *DlXTH23.5* and the TF *DlERF1* was significantly upregulated on different days at 35°C (heat stress) compared with that at 25°C (normal temperature) (**Figure 7B**). *DlXTH25* and the TFs *DlERF5* and *DlWRKY31* were significantly upregulated under high-temperature treatment at 9 and 12 days, and the TF *DlWRKY31* showed a significant downregulation trend under high-temperature stress at 6 days (**Figure 7B**). Notably, *DlXTH23.5/25* and related TFs were all expressed at the highest level under heat stress at 9 days (**Figure 7B**). Interestingly, XET activity and gene expression trends were basically consistent under heat stress. Compared with that of the control, the activity of XET was increased by 4.07-fold at 9 days and 1.57-fold at 12 days (**Figure 7C**). These results showed that the regulatory network composed of *DlXTH23.5/25* and its related TFs might participate in the regulatory pathway of longan under heat stress by regulating the activity of XET.

## Discussion

### The *DlXTH* gene family is evolutionarily conserved and functionally diverse

As important enzymes for cell wall decoration, *XTHs* play important roles in cell wall remodeling *via* cross-linking, construction, and reorganization of xyloglucan (Nishitani and Tominaga, 1992). In our research, 25 nonredundant *XTHs* were identified in “HHZ” longan. Compared with that in other plant species, the number of *XTHs* detected in longan was lower than that in *Litchi chinensis* (29 *LcXTHs*) (Dong et al., 2019), *Arabidopsis thaliana* (33 *AtXTHs*) (Ryusuke and Kazuhiko, 2001), and *Oryza sativa* (29 *OsXTHs*) (Yokoyama, 2004) and higher than that in *Malus sieversii* (11 *MsXTHs*) (Atkinson et al., 2009) and *Prunus avium* (18 *PavXTHs*) (Li et al, 2018). The discrepancy in the number of *XTH* genes between longan and other plant species was related to fewer duplication events in longan. Gene duplication events are a source of diversification of gene function and benefit the expansion of the number of gene family members. In previous studies, WGD was shown to have occurred in *Oryza*, with a large number of gene duplications, and the genome of *Arabidopsis* experienced at least four WGD events (Vision et al., 2000; Thiel et al., 2009). In conclusion, the number of *XTHs* in longan was smaller than that in most other plant species, which may be due to the lack of recent WGD events.

Phylogenetic tree analysis showed that the XTH proteins in longan were more closely related to those in dicotyledons than those in monocotyledons. Although they differed considerably in length, aa, MW, and PI, the group of XTHs in the phylogenetic tree had similar conserved motifs and gene structures, which suggested that *XTHs* of the same group could perform similar functions. According to chromosomal localization and synteny analysis, we found that *XTHs* were unevenly distributed on 10 of the 15 chromosomes of longan, and in the longan genome, 12 segmental duplication events and two tandem duplication gene pairs were found in the *DlXTH* family. Moreover, a group of gene functions was highly conserved in different plant species. Therefore, it is important to precisely identify the true orthologs in other plant species by synteny analysis. In our research, the longan genome had extensive synteny with the *Oryza* and *Arabidopsis* genomes, and 12 and 5 syntenic blocks were identified between the longan, *Oryza*, and *Arabidopsis* genomes, respectively. Compared to the *XTHs* of *Oryza*, more *XTHs* of longan showed a linear relationship with *Arabidopsis*. Therefore, combined with the results of the phylogenetic tree analysis, these data showed that the *XTHs* of longan were more closely related to those of dicotyledons. Because they are essential for the regulation of gene expression, an understanding of *cis*-acting elements inside gene promoters is beneficial to expound the function and regulation of individual genes, which interact with other genes (Hernandez and Finer, 2014). In our research, a number of core promoter elements were identified in the promoter sequences of *XTHs* of longan, which were involved in stress responsiveness (drought, anoxic, low-temperature, defense, and stress), hormone responsiveness (ABA, SA, MeJA, GA, and auxin), light responsiveness, and growth and development. These functional elements explain why the *DlXTH* gene family might play an important role in growth and defense against external stresses *via* regulation by different *cis*-acting elements.

### Transcription factors activate the expression of *DlXTH23.5/25* and are involved in the regulation of longan somatic embryogenesis

The TFs *WRKY* and *AP2/ERF* are among the seven major plant TFs and are important TFs in plants, playing important roles in SE, hormone signaling responses, and stress resistance (Sakuma et al., 2002; Lagacé and Matton, 2004; Zhang et al., 2018; Abbasi et al., 2020). SE is a useful tool during plant reproduction and is the best model for understanding the mechanisms of plant embryogenesis (Guo et al., 2019). Overexpression of *WRKY7* in *Panax ginseng* (Pg) can regulate EC development, and silencing *PgWRKY6* significantly reduced the induction rate of EC. *PgWRKY6* functions upstream of *PgGST*, *PgAPX1*, and *PgSOD* and may mediate auxin-mediated reactive oxygen species (ROS) signaling during SE (Yang et al., 2020). Most *ERFs* are highly expressed in the GE stage of longan SE and are negatively regulated by ethylene (Chen et al., 2018). We selected *DlXTH23.5/25* as the target genes. After using an online tool, 329 TFs were predicted, and we chose three (*DlWRKY31*, *DlERF1*, and *DlERF5*) of 206 TFs that *DlXTH23.5/25* shared based on RNA-seq. We determined the relative expression of *DlXTH23.5/25* and three TFs based on qRT−PCR. The results showed that *DlXTH23.5/25* and three TFs had similar expression trends, and the expression levels of the three TFs gradually increased during the early SE of longan and were high in the GE stage. Therefore, we speculate that the TFs *DlWRKY31*, *DlERF1*, and *DlERF5* can regulate the expression of *DlXTH23.5/25* in the early SE of longan.

The behavior of the TFs *WRKY46* and *MYB15* in the presence and absence of boron (B), which caused the gene expression *CSLB5* and *XTH21*, was similar in both genotypes compared with the wild strain (Col-0) (Gómez et al., 2012). Elongation of *Arabidopsis* hypocotyl cells is regulated by ethylene, auxin, and brassinosteroid signaling, including interactions among *ERF72*, *ARF6*, and *BZR1*. *ERF72* directly interacts with *ARF6* and BZR1 *in vitro* or *in vivo* and antagonizes the regulation of *BEE3* and *XTH7* transcription by *ARF6* and *BZR1* to induce growth of *Arabidopsis* hypocotyls (Liu et al., 2018). In recent studies, increasing evidence has shown that the *XTH* gene family is regulated by the TFs *WRKY* and *ERF* (Liu et al., 2018; Ye et al., 2022). However, no data have been obtained in related studies in longan. To understand how TFs regulate the *XTH* gene family in longan early SE, we preliminarily verified that the TFs *DlWRKY* and *DlERF* could enhance the expression of *DlXTH23.5/25* by a LUC reporter assay performed in tobacco leaves. The results showed that *DlWRKY31*, *DlERF1*, and *DlERF5* enhance the expression of *DlXTH23.5/25* to different extents. To further determine the relationship between *DlXTH23.5/25* and their TFs, the overexpression of the TFs *DlWRKY31*, *DlERF1*, and *DlERF5* was achieved *via* transient transformation into longan ECs and protoplasts. The results confirmed that overexpression of the TFs *DlWRKY31*, *DlERF1*, and *DlERF5* could significantly increase the expression of *DlXTH23.5/25*. Interestingly, the expression of *DlXTH25* was significantly downregulated in longan protoplasts overexpressing *DlWRKY31*. *WRKY* TFs have a variety of biological functions and play important roles in plant growth and development (Yu et al., 2013; Wang and Yu, 2016; Ma et al., 2020) and in response to abiotic (Chun et al., 2020) and biotic stress (Lilly and Subramanian, 2019). Therefore, we speculate that *DlWRKY31* has multiple functions in longan, resulting in different effects on the transcription of *DlXTH25*. The above results further confirmed that the TFs *DlWRKY31*, *DlERF1*, and *DlERF5* could enhance the expression of *DlXTH23.5/25* during early SE and were involved in the regulation of longan SE.

### The transcription factors DlWRKY31, DlERF1, and DlERF5 promote *DlXTH23.5/25* expression to respond to heat stress and increase XET activity

As a characteristic structure of plant cells, the cell wall not only provides structural support for cells but also serves as a line of defense against various pressures (Wang et al., 2019). Therefore, when stimulated by developmental, biotic, or abiotic stimuli, the cell wall can rebuild rapidly (Xu et al., 2020). As cell wall-modifying enzymes, XTHs are involved in many physiological processes. Xu et al. (2020) found that the *AtXTH19* and *AtXTH23* genes were related to salt tolerance in plants. Several *AtXTH* genes were significantly upregulated under aluminum stress (Yang et al., 2011). Overexpression of *CaXTH3* in *Arabidopsis* and tomato improves the drought and salt tolerance of transgenic plants (Cho et al., 2006a; Choi et al., 2011). Transcriptome data from longan ECs under different temperature treatments showed that the expression levels of TFs *(DlWRKY31*, *DlERF1*, and *DlERF5*) and *DlXTH23.5/25* increased significantly under 35°C heat stress. Combined with qRT−PCR data on different days of 35°C high-temperature treatments (6, 9, and 12 days), it was further proven that TFs (*DlWRKY31*, *DlERF1*, and *DlERF5*) and *DlXTH23.5/25* responded to heat stress.

XTH is considered a candidate factor involved in cell wall loosening in plants, and XET activity and *XTH* gene expression are usually correlated with growth (Vissenberg et al., 2000; Vissenberg et al., 2005). The recombinant FvXTH9 and FvXTH6 proteins showed XET activity in strawberries (Witasari et al., 2019). The present finding suggests that the XET activity of *XTH* regulates the degree of growth anisotropy (Van et al, 2007). In our study, the activity of XET increased from the EC to ICpEC and decreased from the ICpEC to GE during early longan SE. In addition, the XET activity of the longan EC increased significantly under heat stress. Previous studies showed that the longan EC could differentiate into the GE at 25°C, while heat stress at 35°C inhibited the differentiation of the EC to GE (Wang, 2019). It is speculated that excessive XET activity may inhibit longan SE. Interestingly, overexpression of *DlXTH23.5/25* and the TFs *DlWRKY31*, *DlERF1*, and *DlERF5* significantly increased XET activity, suggesting that TFs regulate *DlXTH23.5/25* to increase XET activity and participate in cell wall modification. In conclusion, TFs may regulate the activity of XET by regulating the expression level of *DlXTH23.5/25*, thereby regulating the process of SE.

Subcellular localization was confirmed by confocal microscopy of tobacco leaves agroinfiltrated with GFP-tagged versions of DlXTH23.5 and DlXTH25, which localized to vesicles of the secretory pathway and the cell membrane. FvXTH9-YFP and FvXTH6-YFP in strawberry were localized to vesicles and cell membranes of the secretory pathway (Witasari et al., 2019), and AtXTH33 in *Arabidopsis* was localized to the plasma membrane (Ndamukong et al., 2009). Therefore, it was speculated that *DlXTH23.5* and *DlXTH25* may be located on newly secreted xyloglucan and catalyze the transport and transglycosylation of xyloglucan (Thompson and Fry, 2001; Witasari et al., 2019). Therefore, we hypothesized that the regulatory network composed of *DlXTH23.5/25* and its related TFs may be involved in the regulatory pathway of longan under heat stress *via* cell wall repair through the action of XET and that increased XET activity inhibits SE.

## Conclusions

**Figure 8:**
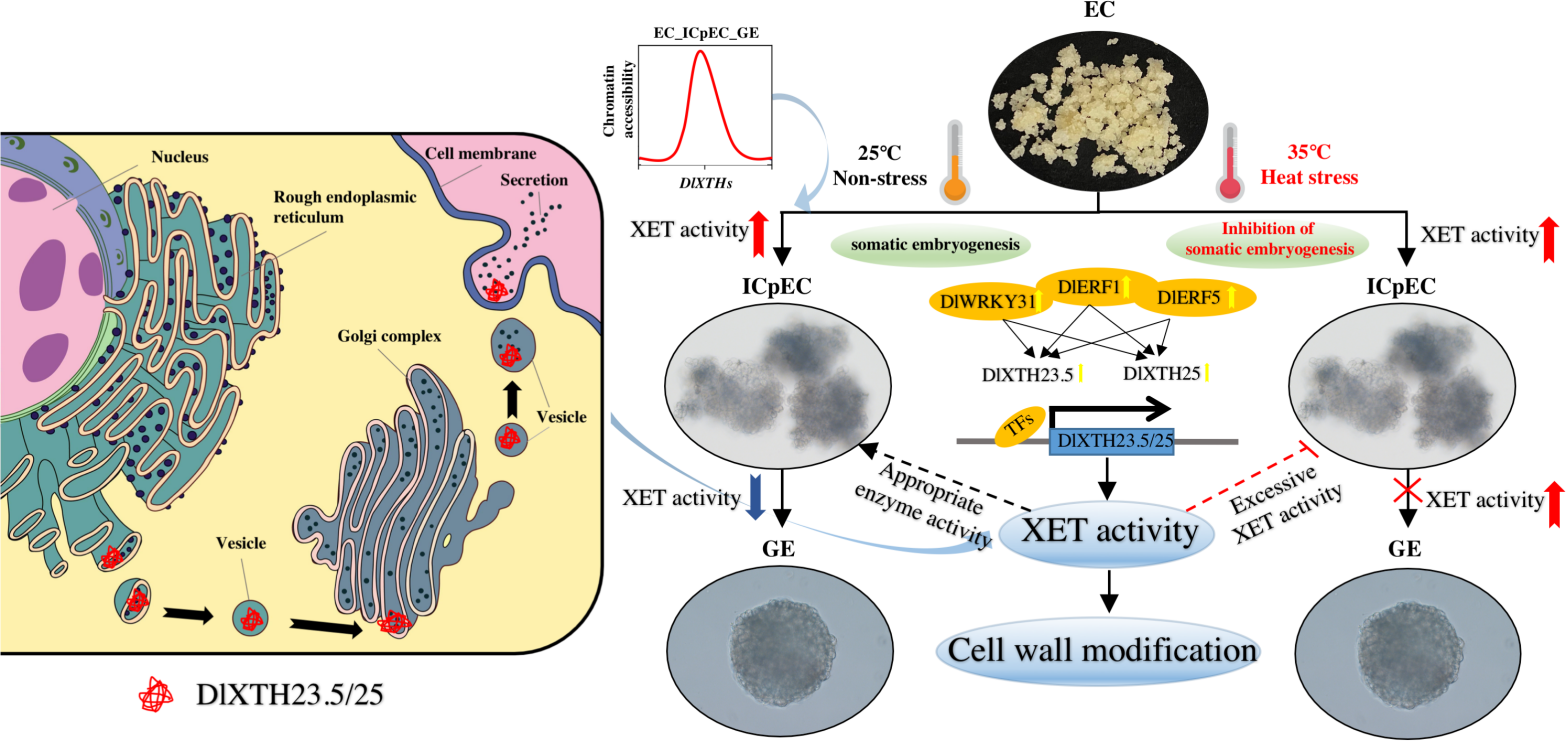
The regulatory network composed of *DlXTH23.5/25* and its related transcription factors may be involved in the regulatory pathway of longan under heat stress *via* cell wall repair through the action of xyloglucan endotransglucosylase (XET), and increased XET activity inhibits somatic embryogenesis (SE). Left: Secretory pathway of the DlXTH23.5/25 proteins in cells. The DlXTH23.5/25 proteins were cotranslationally translocated into the endoplasmic reticulum and subsequently modified in the Golgi. They were transferred *via* a conventional secretory pathway to the plasma membrane and eventually to the cell wall. Right: A model in which transcription factor-mediated regulation of *DlXTH23.5/25* enhances XET activity for participation in cell wall modification during early longan SE and the response to heat stress. Red and blue arrows represent upregulation and downregulation of XET activity, respectively. The yellow arrow represents the upregulation of TF and *DlXTH23.5/25* expression during early longan SE and under heat stress. The dotted line represents possible regulatory mechanisms.

In this study, 25 *XTH* genes were identified in the longan genome. The expression profiles showed that most *DlXTH* genes exhibited specific expression during early longan SE and were widely involved in tolerance to heat stress. Two XTHs, *DlXTH23.5* and *DlXTH25*, were screened at the transcriptional level. Through the study of TFs, it was found that the TFs *DlWRKY31*, *DlERF1*, and *DlERF5* promoted the expression of *DlXTH23.5/25* and increased the activity of XET. At the same time, heat stress not only increased the activity of XET but also significantly increased the expression of TFs and *DlXTH23.5/25*. These findings provide a model in which TF regulation of *DlXTH23.5/25* enhances XET activity to promote cell wall modification during early longan SE and responses to heat stress (**Figure 8**).

## Data availability statement

The datasets presented in this study can be found in online repositories. The names of the repository/repositories and accession number(s) can be found in the article/**Supplementary Material.**

## Author contributions

YL and ZL conceived the project. XM and YC performed most of the experiments. ML and XX performed the genomic expression analysis. XZ and LX prepared the materials. XM analyzed the data and wrote the manuscript. All authors contributed to the article and approved the submitted version.

## Funding

The research was funded by the National Natural Science Foundation of China (31672127), the Natural Science Foundation of Fujian Province (2020J01543), the Constructions of Plateau Discipline of Fujian Province (102/71201801101), and the Technology Innovation Fund of Fujian Agriculture and Forestry University (KFb22022XA and CXZX2019033S).

## Conflict of interest

The authors declare that the research was conducted in the absence of any commercial or financial relationships that could be construed as a potential conflict of interest.

## Publisher’s note

All claims expressed in this article are solely those of the authors and do not necessarily represent those of their affiliated organizations, or those of the publisher, the editors and the reviewers. Any product that may be evaluated in this article, or claim that may be made by its manufacturer, is not guaranteed or endorsed by the publisher.
