# Supplementary figures and images for: Genome-wide analysis of the XTH gene family and functional analysis of DlXTH23.5/25 during early longan somatic embryogenesis
Source: Front Plant Sci. 2022 Nov 23;13:1043464. doi: 10.3389/fpls.2022.1043464 (PMC9727300; doi:10.3389/fpls.2022.1043464)

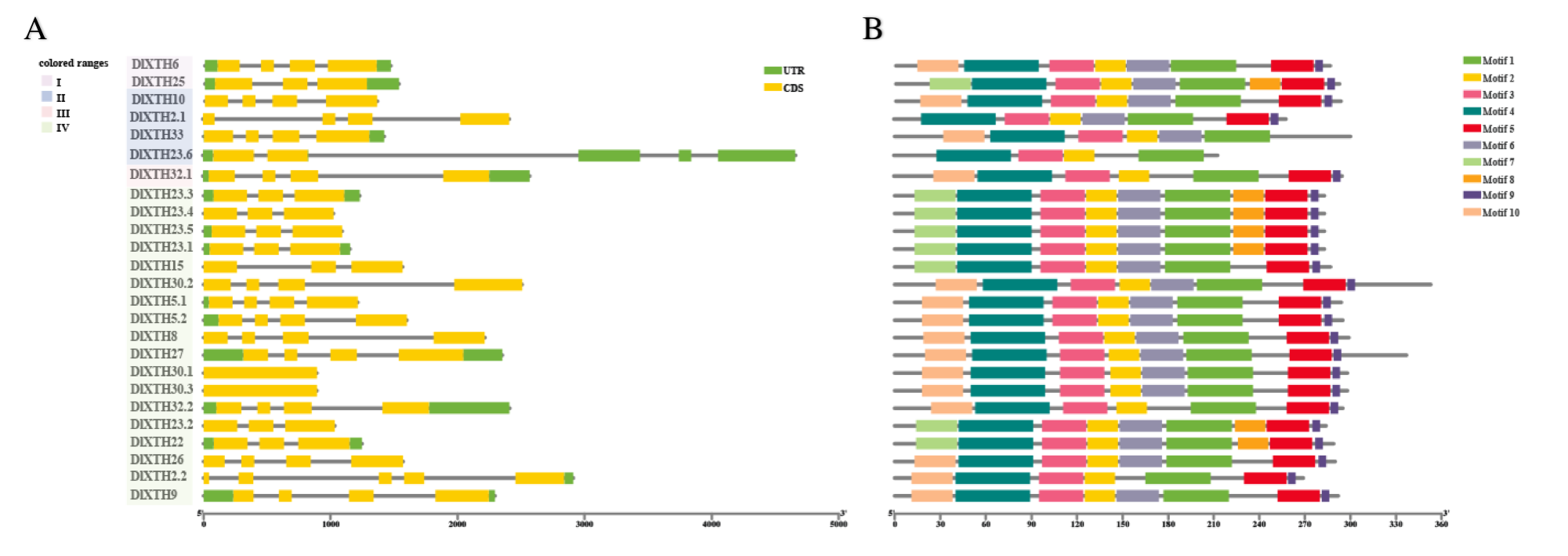

Supplement: Supplementary file 1 [file Image_1.tiff]

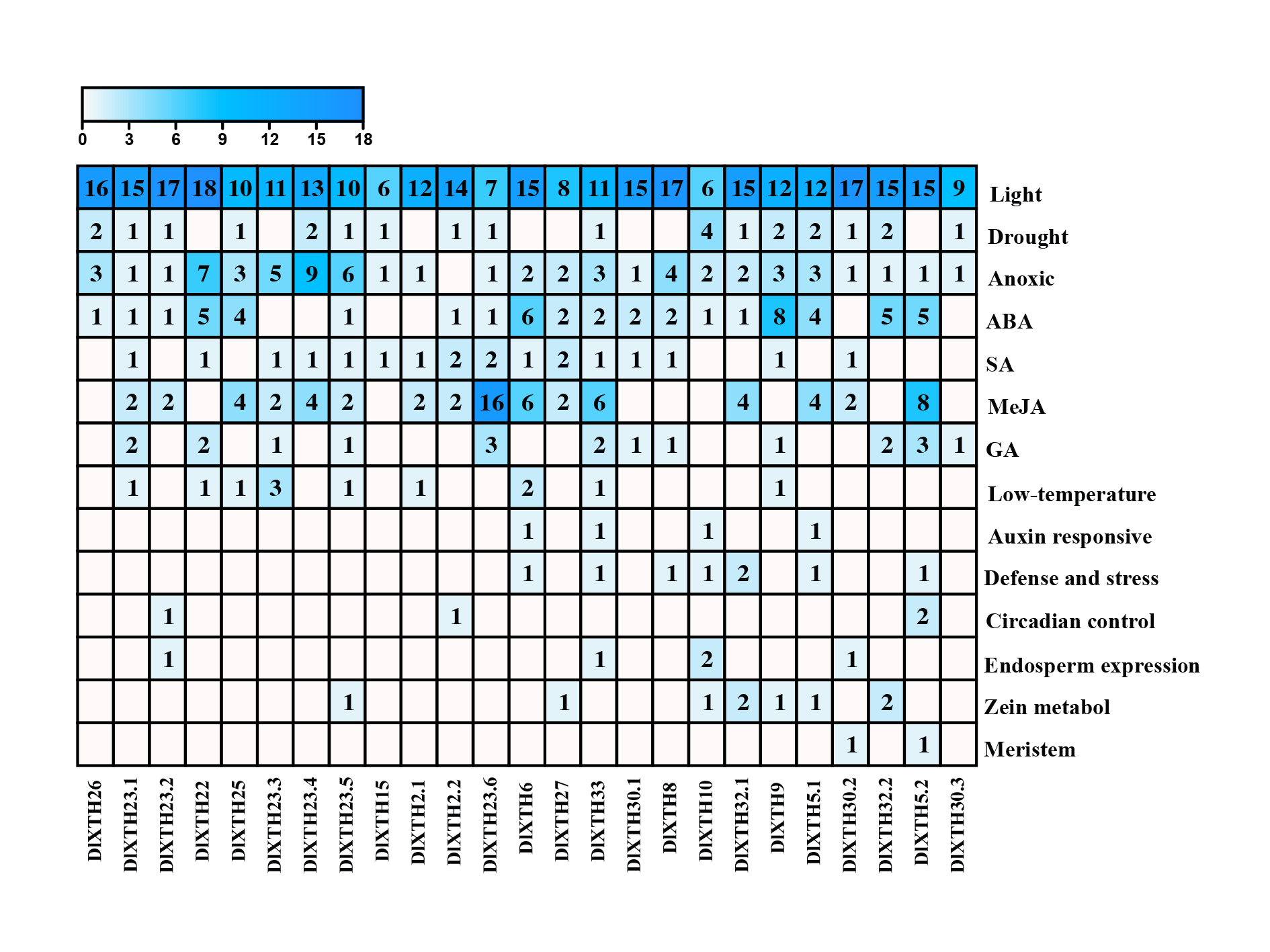

Supplement: Supplementary file 2 [file Image_2.tif]
